# ﻿*Syzygiumtriflorum* (Myrtaceae), a new species from Vietnam

**DOI:** 10.3897/phytokeys.255.143043

**Published:** 2025-04-10

**Authors:** Dang Hung Cuong, Nguyen Thi Kim Thanh, Nguyen Huu Hiep, Dang Ngoc Huyen, Thanh Truong Hoang, Shuichiro Tagane

**Affiliations:** 1 Institute of Tropical Ecology, Joint Vietnam-Russia Tropical Center and Technology Research Center, Hanoi, Vietnam Institute of Tropical Ecology, Joint Vietnam-Russia Tropical Center and Technology Research Center Hanoi Vietnam; 2 Faculty of Biology, University of Science, Vietnam National University, Hanoi, Vietnam Vietnam National University Hanoi Vietnam; 3 Forest Science Institute of Central Highlands and South of Central Vietnam (FSIH), Lam Dong, Vietnam Forest Science Institute of Central Highlands and South of Central Vietnam (FSIH) Lam Dong Vietnam; 4 Kagoshima University Museum, Kagoshima University, Korimoto, Kagoshima, Japan Kagoshima University Kagoshima Japan

**Keywords:** Flora, Indochina, Kon Chu Rang Nature Reserve, Myrtales

## Abstract

*Syzygiumtriflorum* T.T.Hoang, Kim Thanh, S.Tagane & D.H.Cuong, **sp. nov.**, from Kon Chu Rang Nature Reserve, Vietnam, is described and illustrated. The new species is distinguished from related species, *Syzygiumskiophilum*, by its having 4-angled and reddish-grey twigs, oblong leaves with an attenuate apex, less secondary veins of 26–30 pairs, exclusively axillary inflorescences, lanceolate-ovate bracts, sessile flowers, obconical hypanthium (slightly pyriform in flower buds) and more stamens of 18–28. The flowers and fruits were observed in the rainy season, July 2024. This new species grows under a mixed broad-leaved and coniferous forest dominated by *Dacrydiumelatum* and *Dacrycarpusimbricatus*.

## ﻿Introduction

*Syzygium* Gaertn. is the largest genus in the family Myrtaceae, with over 1,200 species primarily found in tropical and subtropical zones. The genus is generally characterised by trees or shrubs, branches sympodial, sometimes 2–4-ridged; leaves simple, opposite, gland-dotted and with intramarginal veins; inflorescences usually paniculate; hypanthium obconic, funnel-shaped or sometimes clavate; calyx lobes and petals 4 or 5; stamens many; ovary inferior; fruits berry (mostly drupaceous) and 1–2-seeded (Hyland 1983; Chantaranothai and Parnell 1994; Pham 2000; Parnell and Chantaranothai 2002; Biffin et al. 2006; Chen and Craven 2007; Govaerts et al. 2008; Craven and Biffin 2010; Ashton 2011; Soh and Parnell 2015).

The genus *Syzygium* in the Indochinese Region was first revised by François Gagnepain under the genus *Eugenia* P. Micheli ex L., identifying 55 species for the Flore générale de l’Indochine (Gagnepain 1914, 1917, 1920). Merrill and Perry (1938) later updated Indochinese *Eugenia* with synopsis, new species and new records and, lastly, transferred *Eugenia* to *Syzygium*. Soh and Parnell (2015) published a revision of Indochinese *Syzygium* (Cambodia, Laos and Vietnam) and enumerated 56 species, based on collections from the 1940s to 2010. Recent research has added 17 new species from Indochina since 2011 (Soh and Parnell 2011; Tagane et al. 2015, 2018; Chantaranothai 2024; Soh et al. 2024; Le et al. 2024), indicating that the diversity of *Syzygium* in the region is still unclarified and there could be more species waiting to be discovered.

In Vietnam, amongst 56 species treated in the most recent taxonomic revision by Soh and Parnell (2015), 49 species were recorded from the country, representing the highest species diversity in Indochina. Since then, eight species have been newly described from Vietnam: *S.honbaense* Tagane, V.S. Dang & Yahara, *S.phamhoangii* Tagane, V.S. Dang & Yahara, *S.yersinii* Tagane, V.S. Dang & Yahara, *S.chantaranothaianum* W. K. Soh & J. Parn., *S.cucphuongense* W. K. Soh & J. Parn., *S.quoctrianum* W. K. Soh, H.V. Sam & J. Parn., *S.samianum* W. K. Soh & J. Parn. and *S.ngheanense* N.S. Ly, N.D. Do & T.H. Le) and three species were recorded for the first time: *S.foxworthianum* (Ridl.) Merr. & L.M. Perry, *S.angkae* (Craib) Chantar. & J. Parn. and *S.thorelii* (Gagnep.) Merr. & L.M. Perry (Soh and Parnell 2011; Tagane et al. 2018; Chantaranothai 2024; Soh et al. 2024; Le et al. 2024). These taxa comprise approximately 60 species that have been inhabiting Vietnam until now.

During a botanical survey and ecological fieldwork conducted in Kon Chu Rang Nature Reserve, Gia Lai Province, Central Highland Vietnam in 2024, the authors collected an unknown *Syzygium* species. After thoroughly comparing this collected sample with available herbarium specimens and reviewing relevant literature, it did not match any previously described species worldwide and we here describe it as a new species, *S.triflorum*.

## ﻿Materials and methods

The specimens of new species were compared with similar species through a literature review, examined from dried specimens from the herbaria, for example, DLU, FOF, HNU, KAG and VNM and online images of the type specimens (downloaded from herbaria, for example, K, P and websites such as JSTOR Global Plants (https://plants.jstor.org/), Tropicos (https://www.tropicos.org/), GBIF (https://www.gbif.org/; https://powo.science.kew.org/), Asian Plant (https://asianplant.net/), Singapore Biodiversity Online Database (https://m.singapore.biodiversity.online/) and Chinese Virtual Herbarium (https://www.cvh.ac.cn/).

The measurement of the description is based on the fresh and dry materials we collected from Kon Chu Rang Nature Reserve. The scientific name and terms follow Hyland (1983), Beentje (2010) and Turland et al. (2018) (Shenzhen code) and the authors mentioned above. The colour photographs are taken under a Canon 600D camera and a Meiji Techno EM-32 stereomicroscope. The plant specimens were deposited at DLU (Da Lat University Herbarium), HNU (VNU University of Science) and the Herbarium of The Joint Vietnam-Russia Tropical Science and Technology Research Center in Ha Noi.

Conservation Assessments are based on the conservation assessments following the recommendations of the IUCN Standards and Petitions Subcommittee (IUCN 2024) and IUCN Red List Categories and Criteria (IUCN 2012).

## ﻿Taxonomic treatment

### ﻿ Syzygium triflorum

Taxon classificationPlantaeMyrtalesMyrtaceae

T.T.Hoang, Kim Thanh, S.Tagane & D.H.Cuong sp. nov.

596A3B02-E4C4-5DDF-A9D1-9B9D0F5DEA03

urn:lsid:ipni.org:names:77360036-1

#### Type.

**Figure 1.**
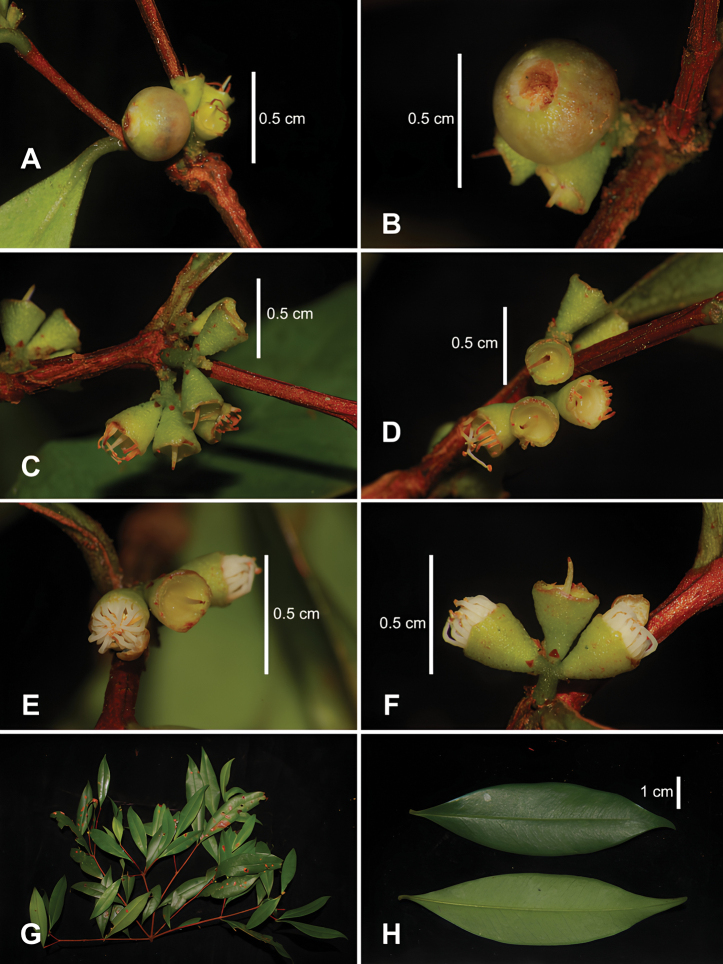
*Syzygiumtriflorum* T.T.Hoang, Kim Thanh, S.Tagane & D.H.Cuong **A** immature fruit in lateral view **B** immature fruits in top view **C** hypanthia in lateral view (after petals and most stamens had fallen off) **D** hypanthia with calyx disc and styles in top view (after petals and most stamens had fallen off) **E** inflorescence in top view, showing flowers before anthesis (left and right) **F** inflorescence in lateral view (3 flowers in cluster) **G** leafy branches **H** leaves, adaxial (upper) and abaxial (bottom) surface. Photos: H. T. Truong and H. C. Dang.

**Figure 2.**
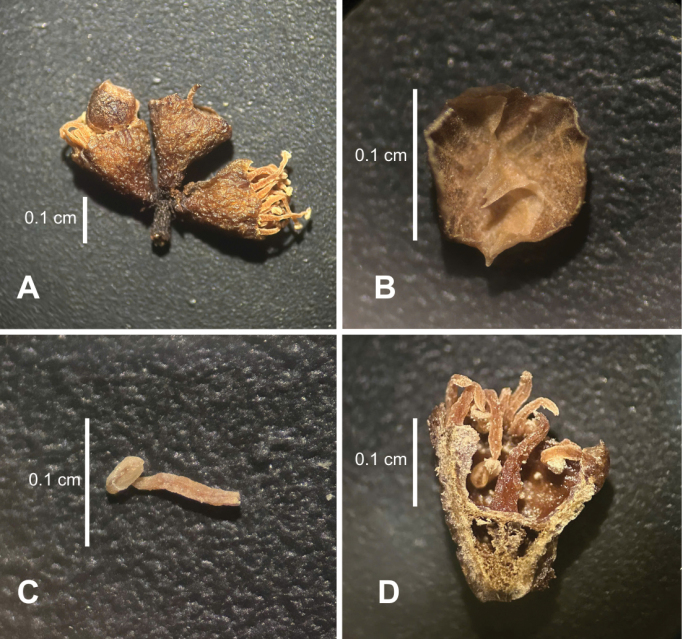
The dried flowers of *Syzygiumtriflorum* T.T.Hoang, Kim Thanh, S.Tagane & D.H.Cuong **A** 3-flowered inflorescence, in lateral view (with an orbicular petal in the left flower) **B** petal, adaxial surface **C** stamen (shorter one) **D** longitudinally dissected flower (with placenta axile). Photos taken by H. T. Truong and H. C. Dang.

Vietnam. Gia Lai Province, K’Bang District, Son Lang Commune, Kon Chu Rang Nature Reserve, in coniferous forest, 200 m from Bai Chay station along the path, 14°28'44.5"N, 108°34'19.2"E, 1015 m a.s.l., 4 July 2024, *T.T.Hoang, N. T. K. Thanh & D.H. Cuong DLU1005* (holotype DLU! isotypes HNU [HNU025528!, HNU025529!], the Herbarium of The Joint Vietnam-Russia Tropical Science And Technology Research Center) (Figs 1, 2).

#### Diagnosis.

**Figure 3.**
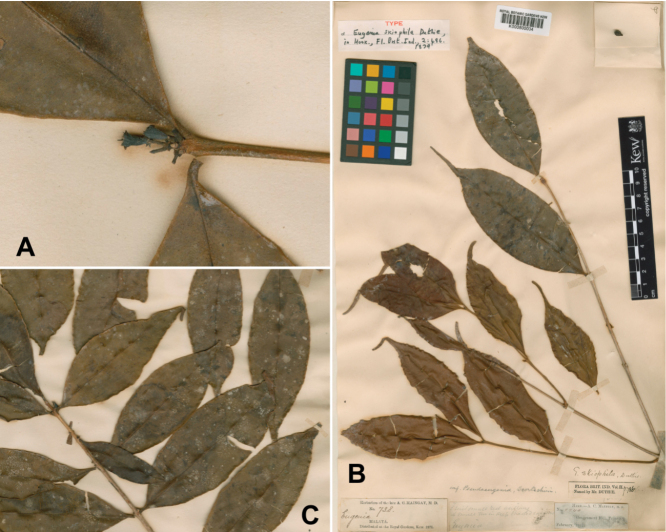
Syntypes of *Syzygiumskiophilum* (Duthie) Airy Shaw **A** hypanthium of *A.C. Maingay 738/2* (K000800033) **B** leaves of *A.C. Maingay 738* (K000800034) and **C***A.C. Maingay 738/2* (K000800033). Image reproduced with permission of © The Board of Trustees of the RBG, Kew. http://specimens.kew.org/herbarium/K000800033; http://specimens.kew.org/herbarium/K000800034.

*Syzygiumtriflorum* could be confused with *S.skiophilum* (Duthie) Airy Shaw distributed in Thailand, Malay Peninsula and Borneo (Airy Shaw 1949; Parnell and Chantaranothai 2002; Ashton 2011). Generally, *S.triflorum* is similar to *S.skiophilum* because they both show small tree habit and short cymose inflorescences with tiny flowers. However, *S.triflorum* differs from *S.skiophilum* in having 4-angled and reddish-grey twigs (vs. terete and blackish-brown in *S.skiophilum*), oblong leaf blades (vs. elliptic-lanceolate, obovate or oblanceolate), fewer secondary veins (26–30 pairs vs. ca. 45 or 14–18 pairs), exclusively axillary inflorescences (vs. temirnal and axillary), lanceolate-ovate bracts (vs. linear), sessile or subsessile flower (vs. with short pseudostalk ca. 1 mm long), obconical hypanthium (slightly pyriform in flower buds) (vs. funnel-shaped) and more stamens (18–28 vs. 8 stamens) (Table 1, Fig. 3).

**Table 1.** Morphological comparison between *Syzygiumtriflorum* and *S.skiophilum.* The charactersistics of *S.skiophilum* was from Parnell and Chantaranothai (2002), Ashton (2011), as well as specimens shown in the Appendix 1.

Parts	*Syzygiumtriflorum* sp. nov.	*Syzygiumskiophilum* (Duthie) Airy Shaw
Twigs	4-angled, reddish-grey	round, dark brown, terete
Leaves	Oblong 7.5–8.5 cm × 2.0–2.2 cm	elliptic-lanceolate, obovate or oblanceolate 7–9 cm × 2.5–3 cm
– Secondary veins	26–30 pairs	ca. 45 pairs or 14–18 pairs
– Apex	attenuate with rounded acute tip 1.5–2 mm long	prominently caudate to caudate-acuminate with slender acumen, ca. 15 mm long.
– Petiole	1–2 mm long	3–6 mm long
Flowers	2.8–3.2 mm × 1.8–2.0 mm	–
– pseudostalks	0–0.5 mm	1 mm
– Colour	yellowish-green	yellowish
– Bracts	lanceolate-ovate, 0.5–0.7 mm long	linear, ca. 3 mm long
– Hypanthium	obconical, 2.0–2.2 mm × 1.8–2.0 mm	ovate-triangular, subacute, 2.8–3.3 mm
– Sepals	triangular, 0.2–0.3 mm long	0.5–0.8 mm long
– Number of stamens	18–28 in 1 whorl	8
Fruits	globose, 4 × 4 mm	ellipsoid, 6 × 4 mm

#### Description.

Trees, 1.5–3 m tall, all part glabrous. Young twigs 4-angled, reddish-grey. Leaves opposite; petioles 1–2 mm long, concave adaxially; blades oblong, 7.5–8.5 cm × 2.0–2.2 cm, dark green and shiny adaxially, pale green, with numerous dark green gland dots abaxially, coriaceous, base cuneate, margin entire, apex attenuate with a rounded acute tip 1.5–2 mm long, mid-rib impressed adaxially, prominent abaxially, secondary veins faint, 26–30 pairs, tertiary veins faintly visible, intramarginal vein 1, 0.6–0.8 mm from the margin. Inflorescences short cymose, axillary or on old branchlets behind leaves, 3–4 mm long, clusters of 3 (rarely reducing to 1) flowers; peduncle 0.8–1 mm long, terete; bracts lanceolate-ovate, 0.5–0.7 mm long, pale green to dark red on the upper half, apex rounded. Flower buds pyriform. Flowers 2.8–3.2 mm × 1.8–2.0 mm, light green, sessile or with pseudostalks to 0.5 mm long; bracteoles triangular, 0.2–0.3 mm long, dark red on the upper half, caducous. Hypanthium obconical, 2.0–2.2 mm × 1.8–2.0 mm, light green. Sepals 4, very broadly triangular, 0.2–0.3 mm long, light green, persistent, apex acute. Petals 4, free, suborbicular, 0.8 mm × 1.0 mm, light green, membranous, concave adaxially. Stamens 18–28 in a single whorl, 0.8–1 mm long, filaments white, anthers ellipsoid, ca. 0.1 mm long, cream-coloured. Ovary 2-locular, many ovules per locule, placentas axile; style and stigma 0.8–1.0 mm long, positioned lower than the stamens. Fruits (immature) globose, 4 × 4 mm, calyx disc convex. Seeds not seen.

#### Flowering and fruiting.

The flowers and fruits were observed during the rainy season in July 2024. At this time, the fruits were not fully matured.

#### Ecology.

This species grows in a mixed broad-leaved and coniferous forest so far known only from Kon Chu Rang Nature Reserve. The forests are dominated by *Dacrydiumelatum* (Roxb.) Wall. ex Hook. and *Dacrycarpusimbricatus* (Blume) de Laub. (Podocarpaceae), along with other angiosperms, such as *Exbucklandiapopulnea*, *Rhodoleiachampionii* (Hamamelidaceae), *Magnoliamediocris* (Magnoliaceae), *Castanopsis* spp. (Fagaceae) and *Schimasuperba* (Theaceae). Many small trees grow in the understorey with *Syzygiumtriflorum* sp. nov., including *Ixora* sp., *Lasianthus* spp. (Rubiaceae), *Eurya* sp. (Pentaphylacaceae) and *Eriobotryabengalensis* (Rosaceae). The habitat receives approximately 60–70% of the total light from the understorey at an elevation around 1,000 m a.s.l.

#### Vernacular.

Trâm ba hoa (three-flowered syzygium), Trâm hoa nhỏ (tiny-flowered syzygium)

#### Etymology.

The species epithet refers to its number of flowers in a inflorescence, which is usually in a cluster of three.

##### ﻿Preliminary conservation status

This species is currently known with only a few populations being found, each consisting of about 30–40 individuals, within a 10-hectare (0.01 km^2^) stand of the mixed broad-leaved and coniferous forest dominated by *Dacrydiumelatum* and *Dacrycarpusimbricatus* at Kon Chu Rang Nature Reserve. The total number of the mature individuals are approximately 200, living in understorey and strictly protected near the Bai Chay ranger station.

The primary natural threat in this area might be random fires, which could cause extreme fluctuations in the number of mature individuals. However, this risk is less prevalent in broad-leaved forests and the mixed broad-leaved and coniferous forests of Kon Chu Rang. Fires have not been recorded in these forests in the Nature Reserve for at least 30 years. Therefore, this species is temporarily regarded as safe in this forest stand.

All five IUCN criteria have been considered, but there is no fully direct or indirect information about the species’ current status or possible threats. Considering the known population is very small, restricted to a 10-hectare stand and the number of mature individuals approximately 200 trees, Endangered (EN) is categorised for the preliminary conservation status of this species according the criteria D (IUCN 2012).

## ﻿Discussion

Although many new taxa have been described over the past two decades and phylogenetic molecular classifications were studied, the classification of *Syzygium* remains complex due to the genus’ megadiverse nature, confusing morphology, misidentified specimens and the lack of morphological data (Biffin et al. 2006; Craven and Biffin 2010; Ahmad et al. 2016; Brambach et al. 2017; Tagane et al. 2018; Widodo and Veldkamp 2021). The morphological characters of fruit structures, seed structures, placentation position and the number of ovules may provide novel insights into evolutionary relationships and the higher systematic classification of *Syzygium* (Briggs and Johnson 1979; Hyland 1983; Biffin et al. 2006), but these characteristics are incomplete in *S.skiophilum.* Therefore, the additional data of the distinctive morphology of *S.skiophilum* and *S.triflorum* sp. nov. may provide evidence of an evolutionary trend to shorter petioles, smaller flowers and one stamen whorl of the genus *Syzygium*.

The short cymes consisiting of three small flowers and single whorl stamens with a tendency to reduce the number of stamens are observed in both *S.triflorum* and *S.skiophilum* in Southeast Asia (Figs 1, 2, 3). The reduction to a single whorl of stamens with tiny stamens occurs in many *Syzygium* species (e.g. *S.chemunjianum*, *S.dhaneshiana*, *S.phamhoangii* and *S.phoukhaokhouayense*) (Shareef et al. 2012; Tagane et al. 2018), but the extreme reduction to a single whorl of stamens along with extremely short cymose is particularly happening in *S.triflorum* and *S.skiophilum.* We suggest that the number of stamen whorls is essential for distinguishing different species and might imply an evolutionary trend within *Syzygium*.

## Supplementary Material

XML Treatment for Syzygium triflorum

## ﻿Additional information

### Author ORCIDs

Dang Hung Cuong https://orcid.org/0000-0003-1208-737X

Nguyen Thi Kim Thanh https://orcid.org/0000-0003-2948-6385

Nguyen Huu Hiep https://orcid.org/0009-0002-4377-3161

Dang Ngoc Huyen https://orcid.org/0000-0001-9742-4675

Thanh Truong Hoang https://orcid.org/0000-0002-8799-5818

Shuichiro Tagane https://orcid.org/0000-0002-1974-7329

### Data availability

All of the data that support the findings of this study are available in the main text

## ﻿Appendix 1

**Specimens examined for comparison with the new species. Malaysia.** Penang, Government Hill, 1871, *Maingay, A.C. 738* (TYPE) (K [K000800034]); 1871, *Maingay, A.C. 738/2* (TYPE) (K [K000800033]); Perak, Nov 1884, *Scortechini 491* (barcode-00071230); Perak, Taiping, *s.n.* (BM [BM000944235]), Benedetto Scortechini; Perak, Larut, *s.coll. s.n.* (K [K000800032]). **Indonesia**. Java, Jan 1910, *s. coll. 52* (K [K000800189]).
